# Folklore and traditional ecological knowledge of geckos in Southern Portugal: implications for conservation and science

**DOI:** 10.1186/1746-4269-7-26

**Published:** 2011-09-05

**Authors:** Luis MP Ceríaco, Mariana P Marques, Natália C Madeira, Carlos M Vila-Viçosa, Paula Mendes

**Affiliations:** 1 Centro de Estudos de História e Filosofia da Ciência (CEHFCi), Palácio do Vimioso, Universidade de Évora, 7000 Évora, Portugal; 2 Conselho de Estudantes de Biologia de Évora (CEBE), Herdade da Mitra, Departamento de Biologia, Universidade de Évora, 7000 Évora, Portugal; 3 Departamento Paisagem, Ambiente e Ornamento (DPAO), Colégio Luís António Verney, Universidade de Évora, 7000 Évora, Portugal

**Keywords:** Gekkonidae, Portugal, reptile conservation, folklore, TEK

## Abstract

Traditional Ecological Knowledge (TEK) and folklore are repositories of large amounts of information about the natural world. Ideas, perceptions and empirical data held by human communities regarding local species are important sources which enable new scientific discoveries to be made, as well as offering the potential to solve a number of conservation problems. We documented the gecko-related folklore and TEK of the people of southern Portugal, with the particular aim of understanding the main ideas relating to gecko biology and ecology. Our results suggest that local knowledge of gecko ecology and biology is both accurate and relevant. As a result of information provided by local inhabitants, knowledge of the current geographic distribution of Hemidactylus turcicus was expanded, with its presence reported in nine new locations. It was also discovered that locals still have some misconceptions of geckos as poisonous and carriers of dermatological diseases. The presence of these ideas has led the population to a fear of and aversion to geckos, resulting in direct persecution being one of the major conservation problems facing these animals. It is essential, from both a scientific and conservationist perspective, to understand the knowledge and perceptions that people have towards the animals, since, only then, may hitherto unrecognized pertinent information and conservation problems be detected and resolved.

## Resumo

*O conhecimento Ecológico Tradicional (CET) e o folclore são repositórios de grandes quantidades de informação sobre a natureza. As ideias das populações locais, percepções e dados empíricos sobre as espécies são importantes fontes de novas descobertas científicas e também para resolver alguns problemas de conservação que possam existir. Procedeu-se à documentação do folclore e do CET que a população do sul de Portugal apresenta sobre osgas, visando compreender principalmente aspectos relacionados com a sua biologia e ecologia, e, também, para documentar o folclore relacionado ao animal. Os resultados sugerem que o conhecimento da população sobre a ecologia e biologia das osgas são precisas e relevantes. Devido às informações prestadas pela população, foi possível ampliar o conhecimento sobre a distribuição geográfica atual da espécie Hemidactylus turcicus, documentando a sua presença em nove novos locais. Além disso percebeu-se que a população ainda possui algumas ideias erradas em que apresentam as osgas como venenosas e portadores de doenças dermatológicas. A presença destas ideias leva a população a ter medo e aversão das osgas, com a perseguição directa a ser um dos principais problemas de conservação que estes animais têm de enfrentar. É muito importante para a ciência e conservação entender o conhecimento e as percepções que as pessoas têm para com os animais, uma vez que as informações pertinentes e problemas de conservação, até então desconhecidos, podem ser detectados e resolvidos*.

## Introduction

Despite its widespread use in many studies, a precise definition of folklore has not yet been established [1]. However, for the purposes of this study it is here defined loosely as a series of legends, music, oral history, proverbs, taboos, jokes, popular beliefs, and customs that are the traditions of a given culture, sub-culture or group, and which have been passed from person to person, generation to generation, by oral transmission or imitation [2]. A variety of sub-types of folklore can thus be distinguished, including human tales, animal tales, trickster tales, etc. In a similar manner, it may be possible to acknowledge the existence of "Folk biology" or an "Ethnobiology" - the popular understanding and categorization of plants, fungi and animals [2] - as a sub-part of a given culture's folklore. Also considered part of cultural folklore, Traditional Ecological Knowledge (TEK) is defined as a cumulative body of knowledge, practice and belief evolving by adaptative processes and handed down through generations by cultural transmission, about the relationship of living beings (including humans) with one another and with their environment [3].

Several recent studies have been published which examine the significance of TEK and folklore, not only in terms of nature conservation, but also as a source of new scientific knowledge [4]. The vast majority have focused on situations in which TEK and folklore play a beneficial role in nature conservation, such as the importance of taboos and social norms for the conservation of species and habitats [5-9], the importance of folklore and the cultural significance of conservation [10,11], and the importance of TEK for science and conservation [4,12-20] However, studies presenting situations in which this type of knowledge has a negative impact on conservation are few. Also few are the studies on ethnoherpetology worldwide. Ethnoherpetology can be defined as a subpart of ethnozoology (which itself can be considered a subpart of ethnobiology), regarding especially the study of the relations and knowledge that people have towards reptiles and amphibians. Worldwide there are few studies on the topic, and mainly concentrated in Africa [21-23], south America [24-28] and Asia [29-32]. In Europe these type of studies are very rare [33-35], and, in Portugal, besides some anecdotal references in some herpetological publications, or in old general ethnographic studies, there are also few studies on the topic [36-38].

We examined the folklore and TEK held by the people of southern Portugal concerning geckos. Our objectives were threefold. Firstly, to search for any possible new information regarding gecko biology and ecology. Secondly, to document local folklore related to the gecko, including any misconceptions held by these communities. Finally, we sought to determine the source of southern Portuguese gecko folklore and TEK, as well as their possible impact on future scientific studies of geckos and their conservation.

### Natural and Cultural History of Geckos and southern Portugal

Southern Portugal is generally considered part of the Mediterranean basin, a biodiversity hot-spot due to the high number of faunal and floral species found there [39]. Following Rivas-Martinez [40], southern Portugal can be biogeographically divided into two main sub-provinces. The Gaditan-Algarvian Sub-province, a lower altitudinal territory mainly characterized by a thermo-mediterranean, dry to subhumid bioclimatic stages [41], and, in contrast, the Lusitan-Extremadurean Sub-province is characterized by thermo- to mesomediterranean, dry to sub-humid bioclimatic stages [41].

The Portuguese continental herpetofauna consists of 28 species of reptiles and 17 species of amphibians [42] that exhibit a wide variety of shapes, colors, behaviors and lifestyles,. The distribution of Iberian herpetofauna is profoundly marked by the differential influence of two major bioclimatic regions: The Atlantic region in the northwest of the Iberian Peninsula, and the Mediterranean region, whose influence is predominant across the rest of the Peninsula [42], including southern Portugal. Reptiles are at home in the dry, warm Mediterranean region, and are thus extremely abundant and diverse. The diversity of reptile species increases from north to south (and from west to east), paralleling aridity gradients [43].

Southern Portugal has a mixed cultural and ethnographic heritage derived from both European and African peoples [44]. From the beginning of the eighth century until the mid-thirteenth century, the south of Portugal was under Arab rule. The legacy of this period appears in the name of the region (*Al-Andalus*), as well as in its culture, architecture and language, with a very large quantity of words, names, techniques and even common practices that still remain today in the Portuguese life. In 1249 A.D., the Portuguese King, Alfonso III finally conquered the kingdom of the Algarve, ending an era of over six centuries of Arab domination [44,45].

**Figure 1:**
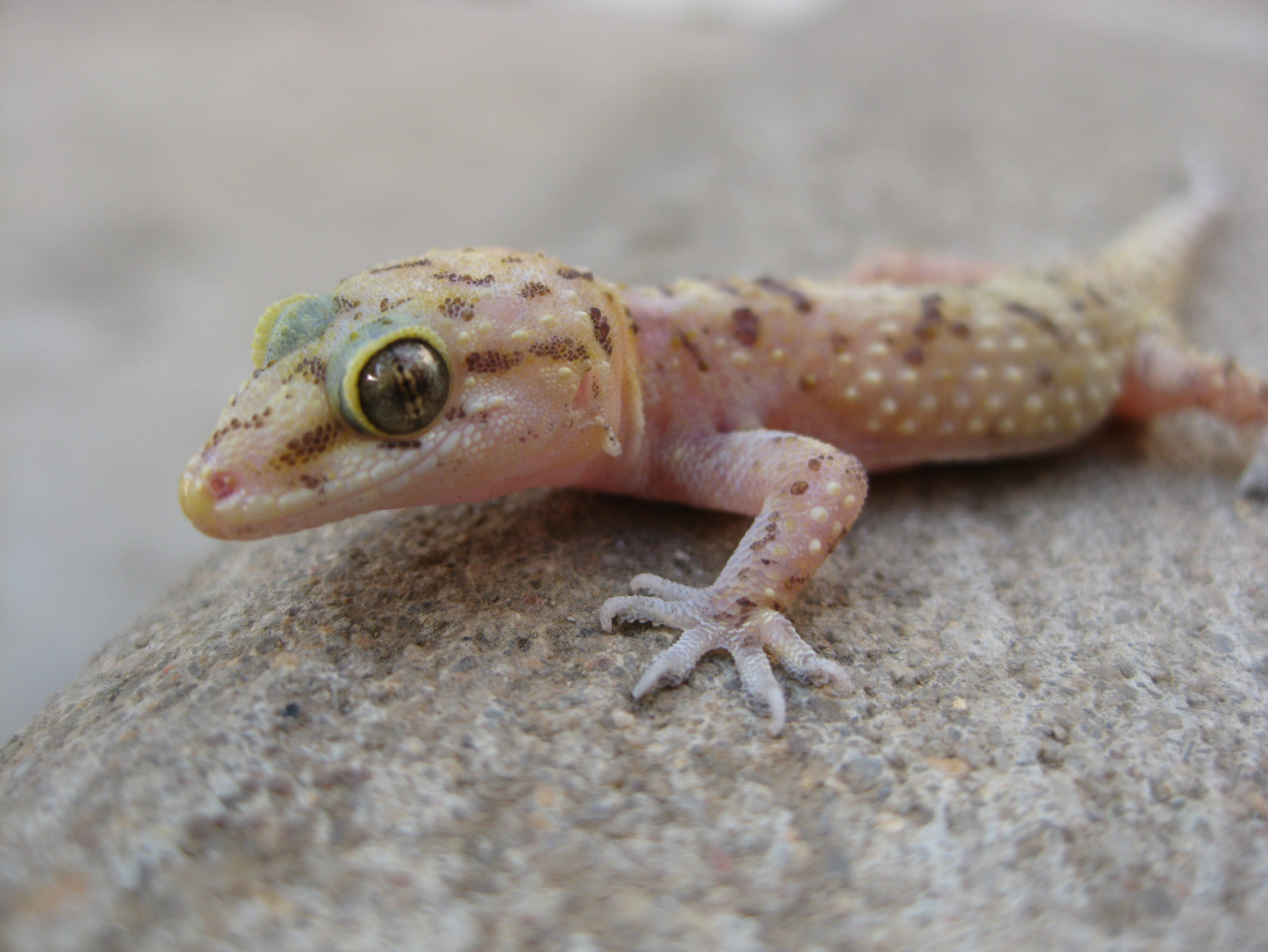
**Mature adult male gecko (*Hemidactylus turcicus*)**. In July 2010 in Mourão, southern Portugal.

Geckos are small reptiles belonging to the Gekkonidae family, and are found in warm climates throughout the world [46,47] (Figure 1). Geckos' toes have a special adaptation that allows them to adhere to most surfaces without the use of liquids or surface tension [48], and as a result they possess the ability to stick to vertical planes, and even upside-down on ceilings and similar horizontal surfaces. Geckos generally have low body volume, large eyes [46,47], and are unique among lizards in their vocalizations, making chirping sounds during social interaction with other geckos [49]. The majority are carnivorous, feeding mainly on invertebrates such as mosquitoes, butterflies and spiders, although some species are able to feed on small vertebrates and even other geckos [46,50]. Two gecko species are currently described for continental Portugal: *Tarentola mauritanica *and *Hemidactylus turcicus *[42]. The latter has a restricted distribution area in Portugal, and is listed as "Vulnerable" according to the Portuguese Vertebrate Red List [51]. Both species are protected by the Portuguese law, under the transposition of Bern Convention on the Conservation of European wildlife and habitats. The most common predators of *H. turcicus *and *T. mauritanica *are snakes, owls, domestic cats, hedgehogs, genets, and rats [50]. Both *T. mauritanica *and *H. turcicus *suffer from human persecution due to public misconception [37,52] while the latter are also probably affected by an ongoing loss and degradation of habitat [51]. There is still also a currently paucity of biological and ecological data regarding both species in terms of their presence in Portugal [42], since few studies were completely dedicated to study these species in the country. *T. mauritanica *and *H. turcicus *may, in certain locations, live sympatrically in open to semi-open landscapes, but are also occasionally found in areas more densely covered by vegetation. Preferred habitats are slopes and stream and river valleys where a multitude of natural and/or artificial crevices provide rocky structures [50,53]. Both species may also be found far from any water bodies, and even deep within human settlements on tree trunks and other vegetative cover [50,53].

Our study aimed to further the conservation of geckos, especially *H. turcicus*, as well as to understand the knowledge and folklore held by local people regarding these animals. Contrary to most folklore and TEK studies, which focus on species found in more rural environments, the present work focuses on a species whose contact with humans occurs mainly in cities and towns. Despite this close contact, these geckos are not appreciated - and are even feared and hated - by the residents of southern Portugal, largely because of pre-existing misconceptions regarding the animals' biological characteristics. The deliberate elimination of geckos is a fairly common phenomenon in the region, but has rarely been studied and is thus not generally understood by the scientific community. The decrease in the gecko population caused by deliberate extermination has not yet been estimated, but may be even greater than the levels observed in the extermination of snakes [38], and as such represents a significant threat.

## Methods

We quantified TEK and folklore through the use of structured, semi-directed interviews of 865 inhabitants (locals) of southern Portugal, of which 517 were women and 348 men. The ages of those questioned ranged from 16 to 98 years, with interviewees coming from the settlements of Évora, Montemor-o-Novo, Reguengos de Monsaraz, Beja, Faro, Albufeira and other nearby localities. Locals were randomly selected in public places. Of the 24 gecko survey sites, 18 were in the Alentejo region and 6 in the Algarve region. *T. mauritanica *is found in all 24 locations [42], while *H. turcicus *is described for only 13.

Our interview survey was developed collaboratively by university ecologists, biologists and sociologists, and included 32 questions that were either open form (respondents expressed their response in their own words) or a combination of open and closed form (multiple choice, but with the opportunity to add comments or additional categories). Survey questions aimed to gather data regarding the distribution, life history, behavior, habitat use, and cultural significance of the gecko, as well as attitudes held by locals towards the animals. These methods were based on those previously employed in similar studies [4,54].

Interviews lasting between 15 and 45 minutes were conducted during the period from 28 September 2010 to 16 February 2011. Interview responses were compiled and summarized as relative percentages of types of response for each question. Informed consent was given by those interviewed.

## Results

The majority of local knowledge of geckos came from oral tradition (55%), direct contact with the animal (50%), and television and internet resources (15%), while only 8% was derived from awareness of scientific literature and 4% contact with biologists. Most local respondents had lived in the Alentejo or Algarve areas since birth, and had a family history in the area extending back at least 2 or 3 generations.

### Gecko Biology and Ecology

**Figure 2:**
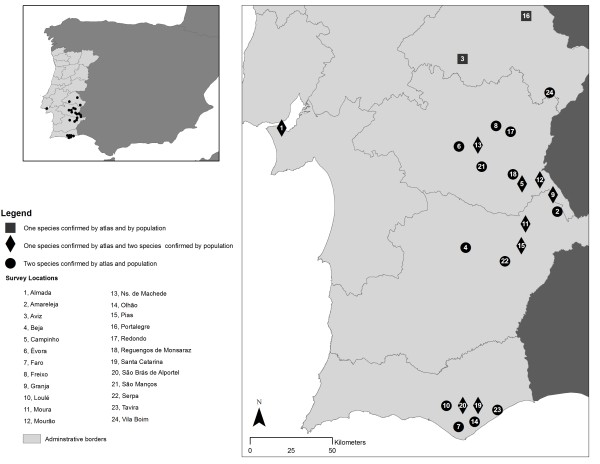
**Distribution of geckos in Portugal at the various survey locations**. For all locations at which only one gecko species is described in the Atlas, the species in question is *Tarentola mauritanica*.

Nearly every local agreed that geckos were reptiles (87%), although some considered them to be amphibians (8%) and even invertebrates (4%). Nearly half of all locals (44%) were able to distinguish between the two gecko species, referring to differences in size, color and rugosity of skin. At 15 of the 24 survey locations, locals recognized the existence of the same number of gecko species described in the *Atlas *[42], but at the other 9 locations pointed to the existence of both species, whereas the *Atlas *[42] described only one (Figure 2).

Nearly every local questioned (98%) named at least one characteristic habitat in which geckos live. The most common answers given were in the walls of buildings (90%), rocks (33%), near lamps (32%), rooftops (28%), on the floor (15%), caves (13%), trees (9%) and bushes (8%). Most locals agreed that geckos like these places because they are warm (22%), safe (18%), and have a food source nearby (24%).

Almost 93% of locals named at least one food item in the gecko diet. The most common food items listed were mosquitoes (82%), spiders (59%), snails (34%) and slugs (34%). Some respondents also stated that geckos eat birds' eggs (4%) and other geckos (3%). Similarly, 86% of locals named at least one predator of geckos, the most common answers being owls (43%), domestic cats (40%), snakes (30%), rats (19%) and bats (14%).

Most locals considered geckos to be solitary animals (84%) that do not show any trace of sociability. Some interviewees recognized that geckos sometimes vocalize (24%), although most (85%) did not know the reason for this behavior. Of those who believed they did, the reasons given were communication with other geckos (8%), mating calls (4%) and defensive behavior (3%).

Nearly all locals referred to geckos as being more active during the summer months (88%) - more precisely during June, July, August and September - with most believing the animals to be more active during these months because of temperature (65%). Most locals also referred to geckos as being more active at night (65%) - more specifically between 9 p.m. and 6 a.m. - although some believed they were active during the morning (8%) and afternoon (13%). Almost half of all locals questioned stated that they were aware of ability of the gecko to attach to walls and other surfaces (51%), with most of these believing geckos to adhere to surfaces via suction cups (40%) or by a type of sticky substance in their feet (8%). Sixty five percent of locals considered the gecko to play an important role in the ecosystem, mainly because they feed on mosquitoes and invertebrates (45%), but also for being the food base of many other animals (34%).

Most interviewees considered the local gecko population to be stable (60%), but 22% considered the population to be decreasing, with the main explanations for this decline being human activity (8%) and climate change (4%).

### Gecko Folklore and Cultural significance

Several locals (4%) thought that geckos feed on human blood and skin, while approximately 25% believed the gecko to be poisonous and 24% that the animal was a vector of dermatological diseases. Several stories were reported regarding the poisonous and disease vector nature of the gecko. One of the most typical stories presented by the locals (10%) related to the poisoning of an entire family by a gecko falling into a saucepan on the stove. "One day, while a woman left the kettle to boil, a gecko snuck by the window without anyone noticing, and fell into the kettle. The woman and her children returned home and drank the coffee without noticing the gecko that had fallen inside. Some hours later, the entire family was very ill and eventually died". A number of variations of the story exist, with some pointing to a pot of soup, milk, or even a teapot instead of a coffee kettle. Others also stated that the family became quite ill, but did not die.

Another typical story reported by 10% of locals concerned the accidental contact between a gecko and human skin. Although the situations in which such contact took place were different, all referred to the result of this contact being the development of skin diseases, rashes, fever and tremendous pain. "Once, a boy was sleeping topless in an old house, and a gecko fell on him while he slept. The next day the boy was full of fever and *cobro*. His skin was red, blistered and sore, and the boy nearly died." Another story related to a bride who put on her wedding dress without noticing that there was a gecko inside, and who then became very ill and full of *cobro*. "*Cobro*" is the name given to a skin inflammation caused by contact with geckos and spiders which can manifest itself in an undefined manner, but generally includes the presence of a gecko- or ring-shaped mark on the torso or limbs of the afflicted, causing his or her death. Others also believed that a gecko falling on someone's head would cause hair loss. Most locals relating these stories believed them to have happened to someone in their towns or villages, or to a distant relative or acquaintance of an acquaintance, but admitted that no such event had ever happened to them personally.

Most locals considered the gecko to be an ugly animal (50%) because of their reptilian, ghostly and transparent appearance. Others (37%) were indifferent to the animal, while a few (13%) considered the gecko to be attractive.

In addition, most locals (55%) considered the gecko as being useful to humans, with many of these believing this to be the case because geckos eat mosquitoes and insects (38%). Ten percent considered the animals to have an important role in the ecosystem. However, most locals (71%) did not feel the presence of geckos in their region to be an asset in terms of the enrichment of their natural and cultural heritage.

### Attitudes towards Geckos

Locals exhibited a variety of attitudes towards the gecko. Most (48%) claimed to ignore the animal when finding one, while 22% kill them, 20% repel the animal, 13% flee in fear and 8% ask another person to kill the gecko. A total of 164 people (19% of the total number surveyed) affirmed that they had deliberately killed one or more gecko in the last 12 months, representing a total of approximately 1092 geckos killed during this period. The most frequently provided reasons for killing geckos were repulsion (42%), fear (14%), and because someone had asked them to kill it (10%). Most locals did not agree with legal gecko protection (71%), with 96% unaware of this legislation altogether.

## Discussion

### Gecko Biology and Ecology

In all cases examined, the interviews carried out here confirm the presence/absence of geckos at the locations refered in the *Atlas *[42]. However, both gecko species were observed by locals at nine of the investigated sites for which only one (*T. mauritanica*) is described in the Atlas, potentially representing nine new records for *H. turcicus *in Portugal. One such case has already been proven after on-site investigation [55]. Since not only did most respondents use very specific characteristics to differentiate the two species (size, color, feet), consistent with the official differences presented in the literature, but also since the climatic and environmental conditions of the new locations are very similar to sites at which the animal has already been described, it is likely that these new locations are correct. This new information represents a significant development, but nevertheless, formal scientific research should be carried out at each site in order to confirm the existence of the species. In any case, the new data collected here may be used to review the conservation status of *H. turcicus*, since the animal has been assigned "vulnerable" status in Portugal because of its reduced distribution [51]. Indeed, this current situation may reflect the lack of studies carried out regarding the species.

Traditional and scientific knowledge of gecko biology and ecology were largely similar (e.g., in terms of taxonomy, diet, predators and habitats), but differed with respect to certain specific aspects - namely the manner of gecko adherence to vertical surfaces. This misconception may be explained by the roundedness of the animals' feet, which often reminded locals of 'suckers'.

### Gecko Folklore and Cultural significance

Unlike that of most other Portuguese amphibians and reptiles, ethnozoological data about geckos is rare. Most ideas and stories reported by respondents in the present study are consistent with those presented by Ceríaco [37], with geckos having very negative connotations and blamed for human skin diseases and poisoning. These beliefs are, however, completely unjustified, since the animal does not possess any kind of toxin that causes poisoning or disease [46]. In addition, there is no known medical or scientific evidence which suggests the gecko to be a vector for the transmission of any kind of bacteria, fungus or virus that may cause dermatological diseases such as the "*Cobro*".

Ceríaco [37] argues that this negative connotation is the result of the region's Arabic cultural heritage, presenting several reasons to support this hypothesis. The influence of Arab culture was felt most strongly in Portugal from the eighth to thirteenth centuries, and left a significant mark on local language, architecture, culture, gastronomy, etc. [45]. As indicated in Ceríaco [37], the noun for gecko in Portuguese (*Osga*) is etymologically and phonetically similar to the Arabic equivalent (*Whazaga*). This idea has been previously outlined in etymological studies which considers "*Osga*" as an arabism in the Portuguese language [56,57].

Similar folklore and stories are shared by the inhabitants of the region stretching from the Asiatic south-west to the Iberian Peninsula and North Africa. For example, the Khushmaan Ma'aza Bedouin tribe from Egypt's Eastern Desert consider geckos to be poisonous, believing contact with the animal leads to death [23]. This tribe also believes the poison of the animal to be contained in its tongue, and that it is transmitted to humans through contact with kitchen utensils or water supply.

Frembgen [29] reports that in Pakistan and northern India very similar stories and ideas to those told by the Portuguese population, especially in terms of the spread of dermatological diseases and the poisoning of food, water or cooking utensils. Communities in northern India and Afghanistan believe that direct contact with geckos is likely to cause skin diseases, and that food is poisoned. In Yemen and many other Arab countries, skin diseases are often attributed to a gecko having run over the face of the afflicted individual as he or she slept (Wranik 1993 in [29]).

By contrast, in countries with only a minor (or entirely absent) Arabic cultural presence, the gecko is seen in a much more positive and friendly light [37]. Even though most people we surveyed considered the animal to be useful to humans - in particular their ability to maintain or reduce the number of mosquitoes - there has as yet been no improvement in the bad reputation of the gecko. This may be due in part to the fact that even though mosquitoes cause humans some discomfort, the incidence of diseases caused by mosquitoes such as malaria is fairly low in Portugal, and the control of mosquitoes by geckos is thus not as important as it is in countries where these diseases are more prevalent.

As an animal considered ugly by most people, their presence is not seen as an asset, either culturally or ecologically. In contrast to smaller and less 'showy' animals, such as reptiles [37,38], invertebrates [58] and even some mammals [59], species such as eagles, pandas, dolphins and the Iberian lynx, on the other hand, are seen as beautiful, interesting and 'fluffy', and serve as flagship species for conservation [60].

### Attitudes towards geckos

Reptiles, as do insects and other animals considered harmful [59-61], tend to suffer from a lack of appreciation by the human population, which translates into less support for their conservation [38]. The situation of geckos in Portugal follows this global trend.

Most inhabitants questioned did not agree that the animal should be legally protected, a view exacerbated by a lack of knowledge regarding the reasons for this legal protection. Although the gecko is protected by law [51], most locals are unaware of this fact, with the animal even facing active persecution. It is likely that even if the population were aware of the law they would act the same way, as there is currently no monitoring undertaken by the authorities. Despite only a small minority of locals partaking in this type of action, such persecution is known to take place with quite considerable frequency. Their proximity to humans only makes it easy to kill the animals on a large scale. With geckos exhibiting very gregarious behavior, low dispersion and having a low number of eggs laid [50,53], the extermination of a substantial group of individuals may lead to significant problems and even the localized extinction of certain populations within the species distribution area.

### Implications for Science & Conservation

Analyzing the differences between TEK and scientific knowledge also represents an important opportunity for conservation research [4]. The information provided by locals made it possible to expand our knowledge of the current geographic distribution of geckos, with the presence of *H. turcicus *reported in locations where it was not previously described (Figure 2), and, in one case [55], leading to the documentation of its presence in a location where it was not previously known. At 15 of the 24 survey locations, locals recognized the existence of the same number of gecko species described in the *Atlas*, but at the other 9 locations pointed to the existence of both species, whereas the *Atlas *described only one (Figure 2). Most respondents described very specific gecko characteristics, and were able to accurately differentiate the two species to a level consistent with the overall differences presented in the scientific literature. The climatic and environmental conditions of the newly described locations are also quite similar to those of the locations at which this animal has already been described. All of these new sites were then investigated in order to confirm the presence of *H. turcicus*, with positive results (not yet published).

This study also discovered a rich local folklore related to geckos. Folklore is a rather complex cultural phenomenon that affects people's lives, their relationship with nature, and even nature itself [3]. Although many authors agree on the necessity of the conservation of folklore [14,62], its persistence may occasionally represent a serious threat to biodiversity, and must therefore be studied, debated, divulgated, and even controlled, by establishing effective and large actions and programs on environmental education and even in the school curricula.

The many myths and folklore tales relating to these animals, in which they are presented as dangerous and venomous [37], contribute to the nature and persistence of public misconceptions held towards them. Ceríaco [38] has argued that the presence of such negative values regarding amphibians and reptiles clearly influences human persecution of these animals. In the case of geckos in Portugal, folklore and misconceptions have had an obviously adverse effect on the relationship between locals and these animals, resulting in their extermination and a lack of public support for their conservation. This persecution has already and will continue to result in the deaths of a considerable number of geckos, and despite the legal protection the animals enjoy, such activity is difficult to police and punish. The problem of direct persecution of herpetofauna is not a residual one, but in fact constitutes a major threat to the survival of some European reptile species, including those not currently endangered [63].

One obvious solution to this problem is to place an increased emphasis on environmental education, as proposed by Whitaker and Shine [64], who suggest that such programs should focus on the clarification of the degree of danger and usefulness of these animals, as well as on the clearer presentation of their real nature (as opposed to their negative portrayal in folklore and their aesthetic characteristics.). Gecko life history, ecology and conservation should also be addressed, with a particular focus on the potential usefulness of these animals as predators of pests, and on their contribution to food-chain equilibrium.

We can therefore conclude that TEK can provide two types of important information: Bio-ecological and cultural. In this study, TEK-derived bio-ecological information led to the report of nine new populations of *H. turcicus*, one of those already proven right [55]. This information is essential in order to review the species' conservation status. Due to its reduced distribution area, *H. turcicus *is currently listed as Vulnerable (VU) in Portugal, although this situation may be due to the lack of studies and information about the species. In contrast, TEK-derived cultural information provided a better idea of the persecution that these animals suffer.

In order to protect animals which are part of a strong cultural heritage and regarding which a large number of stories and misconceptions exist, an interdisciplinary approach is essential. Such an approach includes ethnoherpetological studies, with the analysis of local TEK and folklore, as examination of misconceptions is necessary not only to understand why they still exist in the popular imagination, but also how they may constitute a real risk to the survival of the species in question.

## Competing interests

The authors declare that they have no competing interests.

## Authors' contributions

LMPC designed the investigation, conducted the interviews and wrote all the parts of the manuscript. MPM conducted the interviews and wrote some parts of the manuscript, as also made the statistics of the manuscript. NCM conducted the interviews and wrote some parts of the manuscript, as also made the statistics of the manuscript. CMVV wrote some parts of the manuscript and helped with the map design. PM contributed to the final map. All authors read and approved the final manuscript.
